# Understanding hyaluronic acid induced variation of water structure by near-infrared spectroscopy

**DOI:** 10.1038/s41598-020-58417-5

**Published:** 2020-01-28

**Authors:** Qin Dong, Xueping Guo, Lian Li, Chen Yu, Lei Nie, Weilu Tian, Hui Zhang, Siling Huang, Hengchang Zang

**Affiliations:** 1 School of Pharmaceutical Sciences, Shandong University, Wenhuaxi Road 44, Jinan, 250012 China; 2 Bloomage Biotechnology Corporation Limited, Tianchen Street 678, Jinan, 250012 China; 3 Key Laboratory of Chemical Biology (Ministry of Education), Shandong University, Wenhuaxi Road 44, Jinan, 250012 China; 4 National Glycoengineering Research Center, Shandong University, Binhai Road 72, Qingdao, 266200 China

**Keywords:** Biochemistry, Biological techniques, Biophysics, Biotechnology, Structural biology, Mathematics and computing, Optics and photonics

## Abstract

In order to understand the hydration effect of hyaluronic acid (HA) in aqueous solution, near-infrared (NIR) spectroscopy was used to investigate the HA aqueous solutions at different concentrations and temperature. As HA concentration was raised, there was a nonlinear change in absorption value in the first overtone region of OH, indicating the changes of hydration water. A reconstructed spectrum based on principal component analysis (PCA) was established and analyzed with the concept of aquaphotomics. The results showed that HA acted as a structure maker to make water molecules arranged in order. Water species with two hydrogen bonds (S_2_) and three hydrogen bonds (S_3_) showed the decrease at low concentration range of 0–40 mg/mL, but increased at higher concentration, indicating the difference in water species at different HA concentration. Meanwhile, HA had the ability to improve the thermal stability of water structure, suggesting a potential bio-protective function. This study provides a unique perspective on the molecular interactions between HA and water molecules, which is helpful for understanding the role of HA in life process and may serve as the basis for HA applications.

## Introduction

Hyaluronic acid (HA) is a natural polysaccharide and one of the main component of the extracellular matrix^1^. It is widely distributed in various tissues and organs of animals as well as the capsules of some bacteria, and plays a significant role in life process^1^. Due to its high water retaining capacity, biocompatibility and non-immunogenicity, HA is an attractive material for various cosmetic, food and medical applications^2–4^.

It is known that the structure and morphology of polysaccharides are strongly influenced by water molecules *via* controlling their conformations, various forms of aggregation, thermal properties and kinetics^5,6^. In this respect, the knowledge of the interaction between water and polysaccharide and the hydrated structure is of high importance for understanding its performance in the application. Studies on HA hydration have been reported by using various experimental techniques, including nuclear magnetic resonance (NMR)^7^, viscometry^8^, ultrasonic and densitometry analyses^9^, thermal analysis^10,11^, spectroscopic techniques^12–14^, and by means of computer simulations^15^. A complex relationship between HA and water molecules was partly discovered that the macroscopic properties of HA are significantly dependent on its degree of hydration and the function of HA relies on the hydration capacity^4,16–18^.

Analysis of the hydration water in aqueous solution is challenging, and the structural changes in water induced by HA are yet to be fully understood. Near-infrared (NIR) spectroscopy, as a powerful analytical method, has unique advantages in studies of molecular structure and interaction^19–22^. It mainly reflects the overtones and combination modes of functional groups containing hydrogen atoms, such as CH, OH and NH, which play a significant role in chemical bonding and other chemical phenomena such as hydrogen transfer, hydrogen bonding^23^. The vibrations of these groups greatly reflect even subtle changes in molecular interactions. Therefore, NIR spectroscopy has become a powerful spectroscopic technique in structural biology.

The purpose of this study is to continue and extend the research on the hydration behaviors of HA, but particular attention is paid on investigating the water structure changes induced by HA. NIR spectra of water and aqueous HA solutions with a range of concentration were measured at different temperatures. Principal component analysis (PCA) was conducted on the spectra data to extract the information of different water species. Aquaphotomics was employed to analyze the effect of HA on water structure. The results showed that HA acted as a structure maker to make water structure ordered in aqueous solutions and different water network was promoted at different HA concentrations. Meanwhile, HA had the function of improving the thermal stability of water structures. The results provide new information of HA hydration and possibly shed more light on the function of water in bio-systems.

## Results

### NIR spectra of water and HA solutions

**Figure 1:**
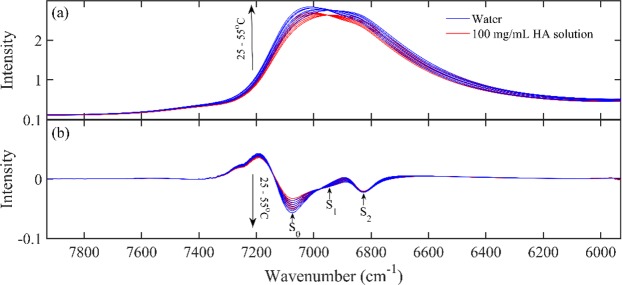
NIR spectra of water, HA aqueous solution (100 mg/mL) from 25 °C to 55 °C (**a**) and the second derivatives of the selected spectra in Fig. 1 (**b**).

Figure 1(a) showed NIR spectra of water, HA aqueous solution (100 mg/mL) from 25 °C to 55 °C. It could be seen the addition of HA caused a decrease in the intensity of the absorption around 6900 cm^−1^, which is an overlap of the absorption mainly due to the combinations of OH symmetric and antisymmetric stretching modes of various water species^24^. Figure 1(b) displayed the second derivatives of the spectra in Fig. 1(a), showing peaks at 7072, 6941 and 6821 cm^−1^. These three peaks can be assigned as S_0_, S_1_ and S_2_, representing water molecules with no, one and two hydrogen bonds and the result was consistent with those reported before^25,26^.

**Figure 2:**
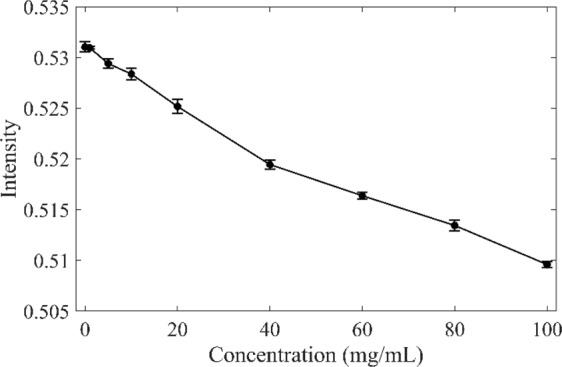
Average absorption of HA solution at 25 °C in the range of 7930–5930 cm^−1^ at different concentrations.

To study the effect of HA concentration on the absorption, the absorption band of 7930–5930 cm^−1^ at 25 °C as the region of the absorption peak between two minima was selected to make further analysis^26,27^. The absorption values in this region were averaged to reduce the noise effect on the data and plotted against HA concentration in Fig. 2. Consistently, the absorption dropped almost linearly when adding HA to water. However, it should be noted that from the concentration of 40 mg/mL onward, the slope starts to decrease slowly.

### PCA analysis on HA concentration dependent NIR spectra

**Figure 3:**
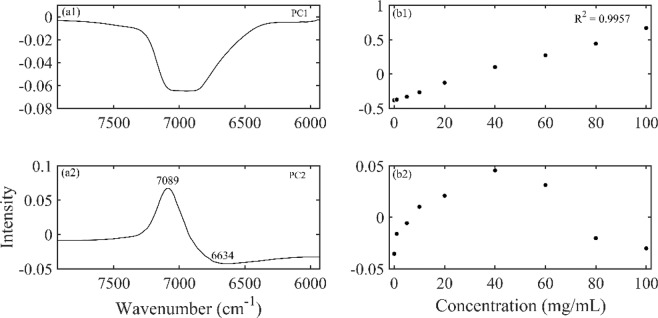
The first two principal components of the spectra of HA solutions with different concentrations at 25 °C extracted by PCA: the loadings (a1, a2), scores (b1, b2). The first principal component accounts for 99.4% of the total variance and the second principal component accounts for 0.53% of the total variance.

To figure out the water molecular structures at different HA concentration, PCA of HA solution spectra had been performed. Figure 3(a),(b) showed the first two loadings and scores of the spectra of HA aqueous solutions, respectively. The first principal component accounted for 99.4% of the total spectral change, and its loading was characterized by a broad negative peak centered at 6900 cm^−1^. With a variance explanation of 99.4%, the first principal component can be considered to explain almost all spectral changes of importance. The second principal component described 0.53% of the total variance, and its loading showed a dominating positive peak at 7089 cm^−1^ and a negative characteristic at 6634 cm^−1^, characterized by less hydrogen bonded OH and more hydrogen bonded OH. The first two principal components had explained 99.93% spectral variation. The score of the remaining principal components fluctuated around zero and could be considered as the change due to noise.

### Analysis of different water species in HA aqueous solutions with different concentration

**Figure 4:**
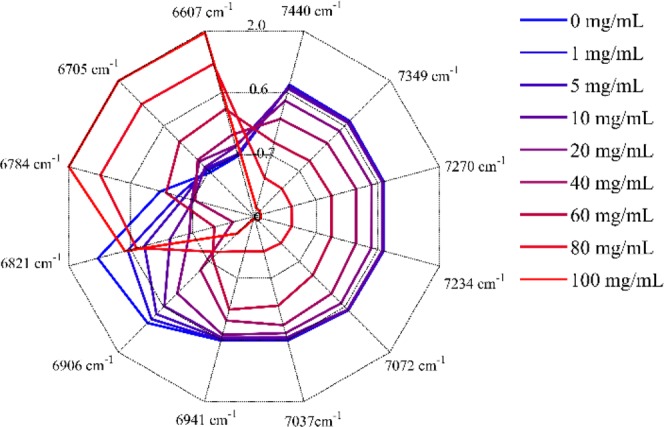
Concentration dependency of water spectral changes depicted by aquagram patterns at 25 °C.

**Figure 5:**
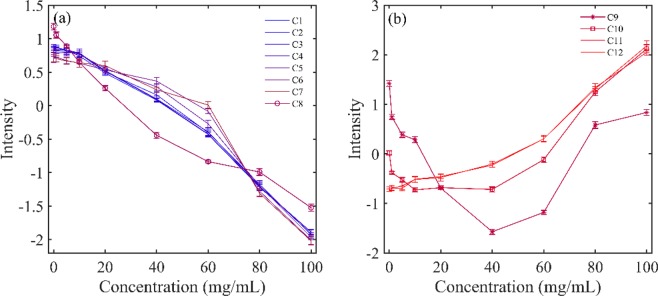
Intensity variation of different water species with different HA concentrations at 25 °C (**a**) for C1-C8 and (**b**) for C9-C12.

In order to further investigate the water structure changes caused by HA, the reconstructed spectra from the first two principal components were calculated. Then, a radar chart named aquagram was established to visualize water spectral pattern at different concentrations. Twelve characteristic water wavenumbers have been defined by Tsenkova *et al*.^28–30^, which cover various conformations of water molecules and can be used to character the spectral patterns in the first overtone region of water. Figure 4 displayed the concentration dependency of water spectral changes depicted by aquagram patterns. Figure 5 showed the intensity variation of each water species with increasing HA concentration. It can be seen that with the increase of HA concentration, the water species of C1-C8 showed a linear downward trend, while the C11 and C12 showed an upward trend. The intensities of C9 (S_2_) and C10 (S_3_) decreased firstly, and when the HA concentration was higher than 40 mg/mL, the intensity began to increase.

### PCA analysis of HA aqueous solution induced by temperature

**Figure 6:**
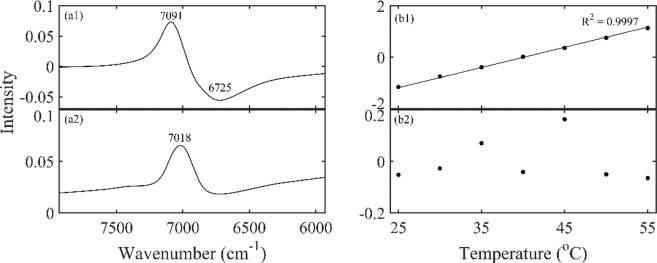
The first two principal components of the spectra of 100 mg/mL HA solutions at different temperatures extracted by PCA: the loadings (a1, a2), scores (b1, b2). The first principal component accounts for 99.8% of the total variance and the second principal component accounts for 0.16% of the total variance.

**Figure 7:**
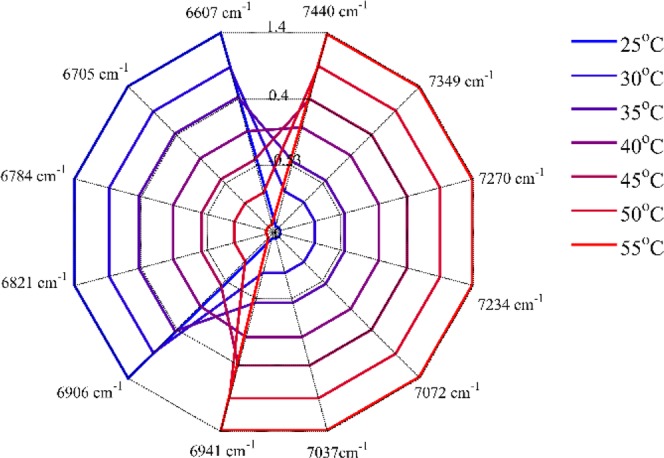
Temperature dependency of water spectral changes depicted by aquagram patterns in 100 mg/mL HA aqueous solution.

In order to further explore the HA effect on water structure, PCA was performed on spectra data of HA solution at different temperatures. Figure 6 showed the first two loadings and scores of the spectra of 100 mg/mL HA solutions from 25 to 55 °C. The first principal component explained 99.8% of the total spectral variance, and its loading was dominated by a positive peak at 7091 cm^−1^ and a broader negative peak centered at 6725 cm^−1^. With a variance explanation of 99.8%, the first principal component can be considered to explain all spectral changes of importance. The second principal component accounted for 0.16% of the total variance. Although its loading showed a dominating positive peak at 7018 cm^−1^, its score vector gave a fluctuation around zero, indicating little more than a noise. Therefore, only the first principal component was used to reconstruct the spectra. Then the reconstructed spectra were depicted by aquagram, as shown in Fig. 7.

### Analysis of different water species in HA aqueous solutions with different temperature

**Figure 8:**
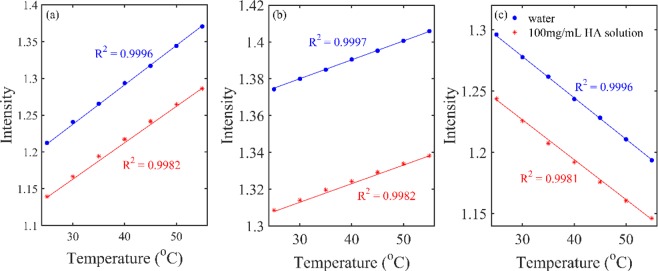
Variation of the peak intensity of S_0_ (**a**), S_1_ (**b**) and S_2_ (**c**) in the reconstructed spectra of water and HA solution (100 mg/mL) with temperature. The solid lines were obtained by linear fitting.

In order to get more information of water structure changes with temperature in the HA solution, the reconstructed spectra from the first component were calculated. Figure 8 showed the intensity variation for the spectral components of S_0_, S_1_ and S_2_, which could be clearly identified in Fig. 1(b). As illustrated in Fig. 8 that, with increasing temperature, the intensity for the spectra of S_0_ and S_1_ increased but that for S_2_ decreased, indicating clearly that more OH of S_0_ and S_1_ was formed but that of S_2_ was dissociated. The result was consistent with the previous studies, that was the larger water clusters dissociated into smaller ones with the increasing temperature^25,26,31^. The temperature effect on S_0_, S_1_ and S_2_ could be obtained by comparing the slope in the Fig. 8. The slope indicates the variation rate of the intensity with temperature, showing the sensitivity of the water species to temperature. The squared correlation R^2^ was obtained from linear fitting between the intensity of the spectral components and the temperature.

## Discussion

The perturbation of hydration water structure due to the presence of HA was investigated by NIR spectroscopy. The decrease in the intensity with increasing HA concentration in Fig. 1 could be explained by the reduction of water. Furthermore, as the temperature increases, the peak position shifts to high frequency which may be due to an increase in weak hydrogen bonded OH and a decrease in strong hydrogen bonded OH^32^. The intensities of the three water species S_0_, S_1_ and S_2_ in Fig. 1(b) changed with increasing temperature, indicating the temperature had the effects on water structure.

The calculation of the absorption values in the region of 7930–5930 cm^−1^ in Fig. 2 proved the nonlinear decreasing behavior of HA aqueous solutions. The reason for the decreasing generally comes from two possibilities. One possibility was that HA absorbed less NIR light than water in the OH stretching region, and therefore, an increase in the concentration of HA would result in a decrease in absorption. It can be considered that although HA has a long chain structure, its contribution to NIR absorption is different from that of pure water. Another possibility was that water molecules were affected by HA, which finally changed the molecular vibration. If the change was entirely caused by the absorption of HA, the absorption value should be linear with HA concentration. Therefore, the changes should also come from the third substance, that was, the contribution of hydration water.

PCA was performed on HA concentration dependent NIR spectra. The score of the first component in Fig. 3 was simply a straight line, suggesting that the spectral changes extracted by the first component varied linearly over the entire concentration range, mainly reflecting the decreasing of water. The score vector was parabolic with a top position of 40 mg/mL, which indicated that the spectral changes captured by the second loading had undergone a transitional change around this concentration. This indicated that the loadings plot of the second principal component reflected the hydrogen bond change of water caused by the increase in HA concentration. Aquagram was plotted based on the reconstructed spectra of PCA in Fig. 4. As a result, quite a characteristic water absorbance pattern was biased towards the low frequency region with increasing HA concentration, indicating the transformation from less hydrogen bonded water structure to more hydrogen bonded water cluster. The intensity variation of each water species in Fig. 5 showed that when the HA concentration was lower than 40 mg/mL, only the formation of S_4_ was promoted. Probably because HA is a long-chain polysaccharide rich in hydroxyl, it can directly combine a large number of water molecules into a tight network structure. The addition of a low concentration of HA may promote the conversion of S_1_, S_2_ and S_3_ into S_4,_ the strongly hydrogen bonded water species. However, as the concentration was further increased above 40 mg/mL, S_4_ continued to increase, while more HA molecules began to promote the formation of S_2_ and S_3_. When HA concentration was lower than 40 mg/mL, the strong hydrogen bonded water structure was preferentially formed and when the concentration continued to rise, the medium hydrogen bonded water network began to form. Moreover, it has been found that in dilute solution, HA behaves as a stiffened random coil, while in concentrated solutions, stiffened random coils show entanglement^33,34^. Therefore, water may be involved in HA entanglement. Previous studies have shown that biomacromolecules normally promote the formation of hydrogen bonds in solution, such as the formation of S_2_, S_3_, and S_4_^22,26^. HA solution exhibits different types of water networks at different HA concentration, which may be related to its long extending structure in water.

PCA analysis was carried out on the spectra of 100 mg/mL HA solutions from 25 to 55 °C.The score of the first principal component in Fig. 6(a) changed almost linearly with the temperature, indicating that the spectra that were extracted by this component changed a linear trend over the whole temperature region. Based on the reconstructed spectra, the aquagram showed quite a characteristic trend that the absorbance was biased towards to high frequency, indicating a dissociation of water cluster and the formation of less hydrogen bonded water structure^32^. The peak intensity of S_0_, S_1_ and S_2_ in Fig. 8 were reduced, which may be due to the volume effect. Meanwhile, the slope R^2^ of S_0_, S_1_ and S_2_ with temperature in the HA solution is less than its slope in pure water, indicating that HA had the effect of improving the stability of water structure and prevent the changes with temperature. The same conclusion was found in the study of other concentrations of HA aqueous solution. Since a large number of hydrogen bonds can be formed between the hydroxyl groups of HA and water^1^, it is speculated that when the temperature rises, the intermolecular hydrogen bonds need to be broken first, which increases the resistance to the vibration of water molecules. This result can be interpreted that the thermal stability of HA solution was achieved by the stability of water^35^ and further provided an evidence of the biological protective function of carbohydrates in aqueous solutions by reducing the sensitivity of water to temperature^36–38^.

## Materials and Methods

### Reagents and sample preparation

HA was provided by Bloomage Biotechnology (Jinan, China) with ${\bar{M}}_{{\rm{w}}}$ of 7775 Da by using gel permeation chromatography coupled with multiple-angle laser light scattering (GPC-MALS). Continuous concentration of HA solutions (1 mg/mL, 5 mg/mL and 10–100 mg/mL with a step of 10 mg/mL) were prepared by dissolving HA powder in double distilled water (Milli-Q, Millipore, resistivity ≥18.5 MΩ cm).

### Spectral measurement

**A**ll NIR spectra were recorded using Antaris II FT-NIR spectrometer (Thermo Fisher scientific Inc., American) equipped with a 1 mm cuvette, tungsten-halogen light source, and InGaAs detector. The spectral range is from 10000 to 4000 cm^−1^ and the spectra are digitalized with 4 cm^−1^ intervals in Fourier transform. The temperature-dependent spectra of water and HA solutions were collected between 25 °C and 55 °C with 5 °C interval. In order to increase the signal to noise ratio, the air reference was measured and subtracted from sample spectrum and the scan number was both set to 32. The spectrum of each sample was measured three times and averaged as a final spectrum.

### Data processing

Spectra were imported into Matlab R2015a (The Math Works Inc., Natick, MA, USA), which was used for data transformation and processing. PCA was performed on spectral data of HA aqueous solution to extract information related to water changes and the reconstructed spectra were calculated accordingly. Then, a radar chart named aquagram was established to visualize water spectral pattern at different concentrations. The ranges of peaks for aquagram depicting were described as follows^28–30^: C1 (7440 cm^−1^: 2 close *ν*_3_: H_2_O asymmetric stretching vibration.), C2 (7349 cm^−1^: OH-(H_2_O)_1,2,4_: water solvation shell.), C3 (7270 cm^−1^: *ν*_1_ + *ν*_3_: H_2_O symmetrical stretching vibration and H_2_O asymmetric stretching vibration.), C4 (7234 cm^−1^: OH-(H_2_O)_1,4_: water solvation shell, O_2_-(H_2_O)_4_: hydrated superoxide clusters, 2 close *ν*_1_: H_2_O symmetrical stretching vibration.), C5 (7072 cm^−1^: water confined in a local field of ions, S_0_: free water, water with free OH-.), C6 (7037 cm^−1^: water hydration band, H-OH bend and OH…O.), C7 (6941 cm^−1^: S_1_: water molecules with one hydrogen bond.), C8 (6906 cm^−1^: OH-(H_2_O)_4,5_: water solvation shell.), C9 (6821 cm^−1^: S_2_: water molecules with two hydrogen bonds, 2 close *ν*_2_ + *ν*_3_: H_2_O bending and asymmetrical stretching vibration.), C10 (6784 cm^−1^: S_3_: water molecules with three hydrogen bonds.), C11 (6705 cm^−1^: S_4_: water molecules with four hydrogen bonds.), C12 (6607 cm^−1^: *ν*_1_: H_2_O symmetrical stretching vibration, *ν*_2_: H_2_O bending vibration, strongly bound water.).

Twelve characteristic water absorption bands mentioned above covering the most unique water species termed as water matrix coordinates (WAMACS) were used as axes and the values for aquagram were derived from the following equation^39^:

1 $${A}_{\lambda }^{^{\prime} }=({A}_{\lambda }-{\mu }_{{\rm{\lambda }}})/{\sigma }_{\lambda }$$

where *A*_*λ*_, *μ*_*λ*_ and *σ*_*λ*_ are absorbance after multiplicative scatter correction (MSC), the average of all spectra and the standard deviation of all spectra, respectively, at wavelength *λ* (convert to wavenumber in this paper).
